# ATAC-seq reveals alterations in open chromatin in pancreatic islets from subjects with type 2 diabetes

**DOI:** 10.1038/s41598-019-44076-8

**Published:** 2019-05-23

**Authors:** Madhusudhan Bysani, Rasmus Agren, Cajsa Davegårdh, Petr Volkov, Tina Rönn, Per Unneberg, Karl Bacos, Charlotte Ling

**Affiliations:** 1 Epigenetics and Diabetes Unit, Department of Clinical Sciences, Lund University Diabetes Centre, Lund University, Scania University Hospital, Malmö, Sweden; 2 Department of Biology and Biological Engineering, National Bioinformatics Infrastructure Sweden, Science for Life Laboratory, Chalmers University of Technology, Göteborg, Sweden; 3 Department of Cell and Molecular Biology, National Bioinformatics Infrastructure Sweden, Science for Life Laboratory, Uppsala University, Uppsala, Sweden

**Keywords:** Type 2 diabetes, Genetics research

## Abstract

Impaired insulin secretion from pancreatic islets is a hallmark of type 2 diabetes (T2D). Altered chromatin structure may contribute to the disease. We therefore studied the impact of T2D on open chromatin in human pancreatic islets. We used assay for transposase-accessible chromatin using sequencing (ATAC-seq) to profile open chromatin in islets from T2D and non-diabetic donors. We identified 57,105 and 53,284 ATAC-seq peaks representing open chromatin regions in islets of non-diabetic and diabetic donors, respectively. The majority of ATAC-seq peaks mapped near transcription start sites. Additionally, peaks were enriched in enhancer regions and in regions where islet-specific transcription factors (TFs), e.g. FOXA2, MAFB, NKX2.2, NKX6.1 and PDX1, bind. Islet ATAC-seq peaks overlap with 13 SNPs associated with T2D (e.g. rs7903146, rs2237897, rs757209, rs11708067 and rs878521 near *TCF7L2*, *KCNQ1*, *HNF1B*, *ADCY5* and *GCK*, respectively) and with additional 67 SNPs in LD with known T2D SNPs (e.g. SNPs annotated to *GIPR*, *KCNJ11*, *GLIS3*, *IGF2BP2*, *FTO* and *PPARG*). There was enrichment of open chromatin regions near highly expressed genes in human islets. Moreover, 1,078 open chromatin peaks, annotated to 898 genes, differed in prevalence between diabetic and non-diabetic islet donors. Some of these peaks are annotated to candidate genes for T2D and islet dysfunction (e.g. *HHEX*, *HMGA2*, *GLIS3*, *MTNR1B* and *PARK2*) and some overlap with SNPs associated with T2D (e.g. rs3821943 near *WFS1* and rs508419 near *ANK1*). Enhancer regions and motifs specific to key TFs including BACH2, FOXO1, FOXA2, NEUROD1, MAFA and PDX1 were enriched in differential islet ATAC-seq peaks of T2D versus non-diabetic donors. Our study provides new understanding into how T2D alters the chromatin landscape, and thereby accessibility for TFs and gene expression, in human pancreatic islets.

## Introduction

Genetic, epigenetic and environmental factors contribute to development of type 2 diabetes (T2D)^1,2^. Genome-wide association studies (GWAS) support islet dysfunction to be a key defect in T2D^3^. A large proportion of T2D-associated SNPs are located at regulatory regions and enhancer elements which control gene transcription^4^. Both the transcriptome and methylome are altered in T2D islets^5–7^. However, additional studies are needed to fully dissect the molecular mechanisms contributing to islet dysfunction. Importantly, a map of the open chromatin has not been generated in pancreatic islets of numerous subjects with T2D.

Transcription factors (TFs) bind to DNA in a sequence-specific manner by removing or moving the nucleosomes to form open chromatin^8^. These open chromatin or nucleosome free regions are sensitive to nuclease enzymes. Chromatin immunoprecipitation (ChIP)-seq and DNase-seq studies from ENCODE show that open chromatin regions include all classes of cis-regulatory elements (cis-REs) e.g. promoters, enhancers and insulators^9,10^. Studies performed in human islet cells revealed that T2D-associated loci reside at open chromatin regions and enhancers^11–15^. However, these studies were mainly performed in islets/cells of non-diabetic donors and knowledge of how T2D alters the open chromatin structure in islets is needed.

To map the open chromatin landscape in relation to T2D, we performed assay for transposase-accessible chromatin using sequencing (ATAC-seq) in human pancreatic islets from T2D and non-diabetic donors. ATAC-seq is a sensitive recently developed method with ability to map open chromatin in a small number of cells^16^. We further integrated our human islet ATAC-seq data with RNA-seq and published islet ChIP-seq data of histone modifications and TFs.

## Methods

### Islets

Human islets of 9 non-diabetic donors and 6 donors diagnosed with T2D were obtained from the Nordic Network of Islet Transplantation, Uppsala University, Sweden. Donor characteristics are presented in Table 1. Donor or his/her relatives had given their written consent to donate organs for biomedical research upon admission into the intensive care unit. The work was approved by ethics committees at Uppsala and Lund Universities. Islets were isolated and cultured as described^6^. All islet preparations used in this study had a purity above 80%. Fresh islets were picked with pipette under stereo microscope and snap frozen in aliquots (~30 islets) (n = 5 donors) or immediately used for ATAC-seq (n = 5 donors). Frozen islets were obtained from our biobank and were thawed on ice (n = 5 donors). This information is presented in Supplemental Table 1 and there are T2D donors in all three categories. 1 μl of islet pellet (corresponding to ~30 islets) was used for each ATAC-seq experiment.

**Table 1:** Characteristics for donors of pancreatic islets included in the ATAC-seq analysis.

	Non-diabetic donors (n = 9)	T2D donors (n = 6)	P-value
Sex (M/F)	6/3	2/4	
Age (years)	61.4 ± 14.5	61.8 ± 7.0	0.58
BMI (kg/m^2^)	26.4 ± 3.3	30.3 ± 2.3	0.049
HbA1c (mmol/mol)	35.3 ± 3.6*	49.2 ± 8.8**	0.002
HbA1c (%)	5.37 ± 0.3*	6.66 ± 0.8**	0.002

Data presented as mean +/− SD. Data analyzed by Mann-Whitney *U*-test *Data available for seven donors. **Data available for five donors.

### ATAC-seq

ATAC-seq was performed as previously described^16^ in our human islets (see Supplemental material and methods).

### Analysis of ATAC-seq data

See Supplemental material and methods.

### Annotation of ATAC-seq peaks

The ATAC-seq peaks were annotated to genomic elements such as transcription start sites (TSS), transcription termination sites (TTS), exons and introns using GENCODE version19 for GRCh37. Each peak was annotated in relation to all these elements and may thereby have multiple gene annotations.

### Islet RNA-seq

See Supplemental material and methods.

### TF motif analyses of ATAC-seq peaks

We performed two different TF analyses of the ATAC-seq data; (i) looking at overlap between islet ATAC-seq data and binding of specific TFs in human islets using ChIP-seq data generated by Pasquali *et al*.^11^ and (ii) looking at overlap between islet ATAC-seq data and putative TF binding motifs. TF binding motif analysis of ATAC-seq data was performed using HOMER v4.7.2^17^. Only known motifs from HOMER’s motif database were considered. We studied motifs enriched in ATAC-seq peaks of non-diabetic donors, and motifs enriched in T2D peaks relative non-diabetics. The latter analysis was performed by *findMotifsGenome*.*pl* with the peak set for non-diabetics as background.

### Overlapping ATAC-seq peaks with public ChIP-seq datasets

See Supplemental material and methods.

### Lift-over of published datasets

UCSC lift-over online tool (https://genome.ucsc.edu/cgi-bin/hgLiftOver) was used to convert the original assemblies of published datasets into GRCh37/hg19 using BED files or chromosome coordinates.

### Functional experiments in beta-cell line

INS-1 832/13 rat beta-cells were used in functional experiments as described^6^. The siRNAs used for knockdown were s144488 (siGabra2) and s132130 (siSlc16a7) and a custom negative control siRNA (5′-GAGACCCUAUCCGUGAUUAUU-3′) (siNC). Knockdown was verified with qPCR and TaqMan assays for *Gabra2* (Rn01413643_m1) and *Slc16a7* (Rn00568872_m1). Assays for *Hprt1* (Rn01527840_m1) and *Ppia* (Rn00690933_m1) were used as endogenous controls and knockdown was calculated with the geometric mean method. Insulin secretion was analyzed as previously described^18^, except that insulin was determined with an ELISA (Mercodia, Uppsala, Sweden). All siRNA and TaqMan assays were ordered from Thermo Fisher Scientific, Waltham, MA, USA.

### Statistics

Mann-Whitney *U*-tests were used for clinical data. Fisher’s exact tests were used to test if the number of ATAC-seq peaks were significantly associated with T2D and gene expression. Enrichments were analyzed by chi-square tests and a 5% overlap was considered by chance, i.e. 5% of the total number of peaks for each group. False discovery rate (FDR) analyses were used to correct for multiple testing.

Fisher’s exact test was used for an occupancy-based analysis (i.e. to identify islet ATAC-seq peaks that were found in significantly more non-diabetic compared to T2D donors or vice versa). The R Diffbind package and edgeR package^19^ were used for an affinity-based analysis (i.e. to identify islet ATAC-seq peaks where the mean number of mapped reads differ between the groups). Gender and sample treatment (frozen/non-frozen) were included as covariates in the design matrix. The whole ATAC-seq workflow was implemented in Snakemake^20^. qPCR and insulin secretion experiments performed in beta-cells were analyzed with the non-parametric Mann-Whitney test.

### Ethics

Informed consent for organ donation for medical research was obtained from pancreatic donors or their relatives in accordance with the approval by the regional ethics committee in Lund, Sweden (Dnr 173/2007). This study was performed in agreement with the Helsinki Declaration.

## Results

### Mapping the open chromatin landscape in human islets

**Figure 1:**
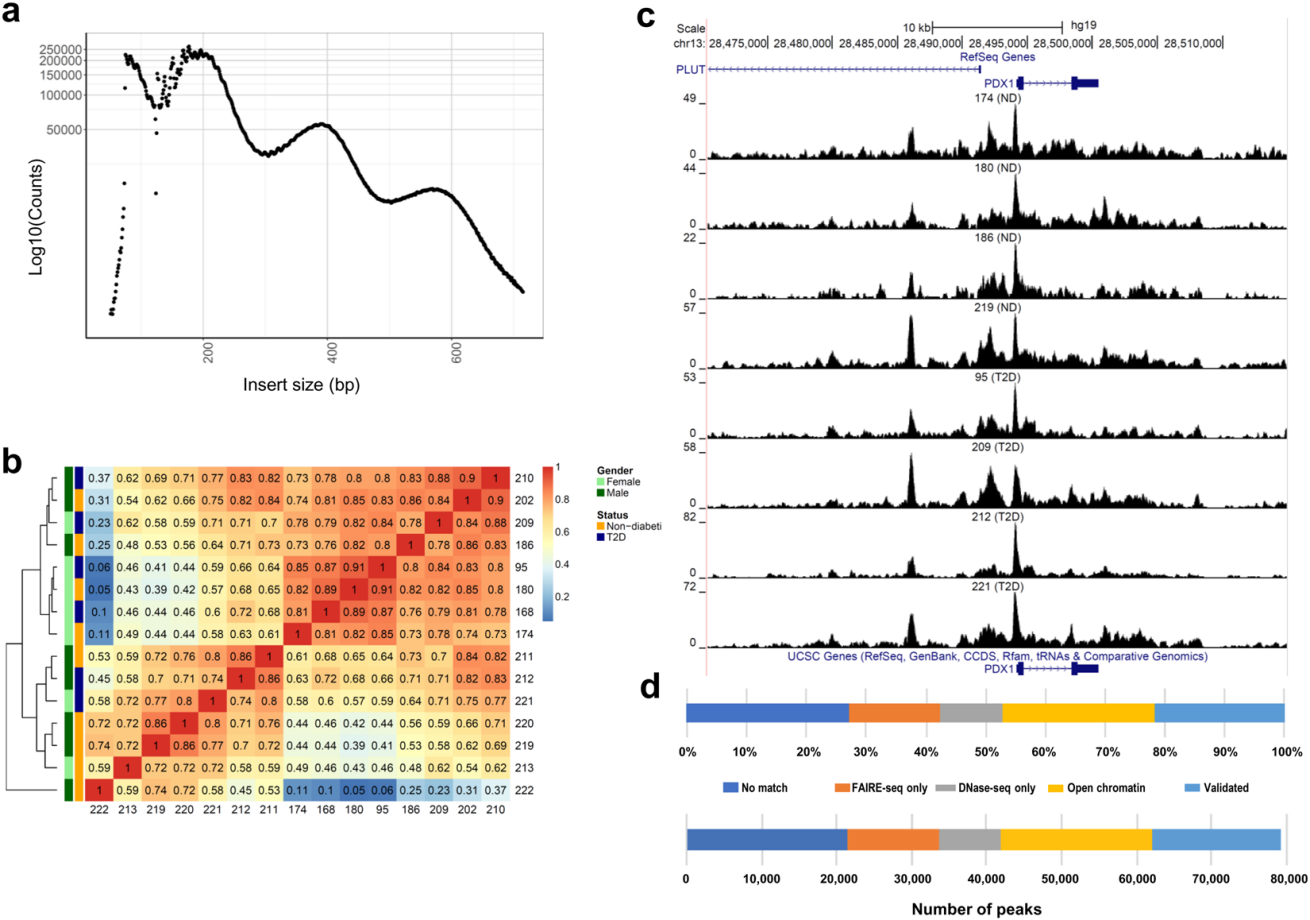
(**a**) Insert size distributions of islet ATAC-seq data showing clear nucleosome phasing. The first peak represents the open chromatin, peak 2 to 4 represent mono-, di- and tri-nucleosomal regions. (**b**) Hierarchical clustering of the Spearman correlation of the ATAC-seq data, as calculated by binning reads for consecutive bins of 10 kilobases including ATAC-seq data of all analyzed islet samples and excluding Y-chromosome data. (**c**) Representative sequencing tracks for the *PDX1* locus show distinct ATAC-seq peaks at the promoter and the known enhancer in human islets. The ATAC-seq data have been normalized to take sequencing depth into account and the scale on the y-axis was chosen for optimal visualization of peaks for each sample. (**d**) Proportions of islet ATAC-seq peaks identified in at least three donors (79,255 open chromatin peaks) overlapping with ENCODE open chromatin data generated using FAIRE-seq and DNaseI-seq in human islets. ATAC-seq peaks overlap with the following number and categories of ENCODE peaks: 17,172 validated peaks, 20,178 open chromatin peaks, 8,288 DNaseOnly peaks and 12,063 FAIREonly peaks. 21,554 ATAC-seq peaks did not match with ENCODE peaks.

We performed ATAC-seq to map the open chromatin accessible regions in human islets of 15 donors (6 with and 9 without T2D). The characteristics of these donors are presented in Table 1. The ATAC-seq libraries were sequenced to an average of 101.3 million reads per islet sample. The quality of the ATAC-seq data was high for all islet samples with expected fragment distribution and clear nucleosome phasing (Fig. 1a and Supplemental Fig. 1a–d). Strand cross-correlation statistics analyzed by phantompeakqualtools support high quality ATAC-seq data (Supplemental Table 1). Figure 1b shows hierarchical clustering of Spearman correlations of the samples, as calculated by deepTools. Islet ATAC-seq data correlated between the donors (Fig. 1b).

ATAC-seq peaks were only considered if they exist in at least three islet samples. Using this cut-off, 79,255 open chromatin peaks were identified when we combined ATAC-seq data from non-diabetic and diabetic islet donors (Supplemental Table 2). Using the same criteria but looking at non-diabetic and T2D donors separately, we identified 57,105 and 53,284 open chromatin regions in respective group (Supplemental Tables 3 and 4). Some sequencing ATAC-seq tracks annotated to *PDX-1*, a transcription factor regulating beta-cell development and insulin gene expression^21^, and *FOXA2*, a regulator of beta-cell development, are presented in Fig. 1c and Supplemental Fig. 2a,b.

We next compared our islet ATAC-seq data (79,255 open chromatin peaks) with ENCODE open chromatin data generated using FAIRE-seq and DNaseI-seq as well as with ATAC-seq data generated by Thurner *et al*. in human islets^9,12,22^. Approximately 73% of our ATAC-seq peaks overlap with ENCODE peaks (peaks identified by FAIRE-seq and DNaseI-seq in islets of non-diabetic donors, https://www.ncbi.nlm.nih.gov/geo/query/acc.cgi?acc=GSM1002652) further supporting the quality of our data (Fig. 1d). The overlap between our ATAC-seq peaks and ENCODE data is also presented in Supplemental Tables 2–4. Moreover, 79,193 of our ATAC-seq peaks (99.9%) overlapped with data generated by Thurner *et al*. (Supplemental Table 5).

**Figure 2:**
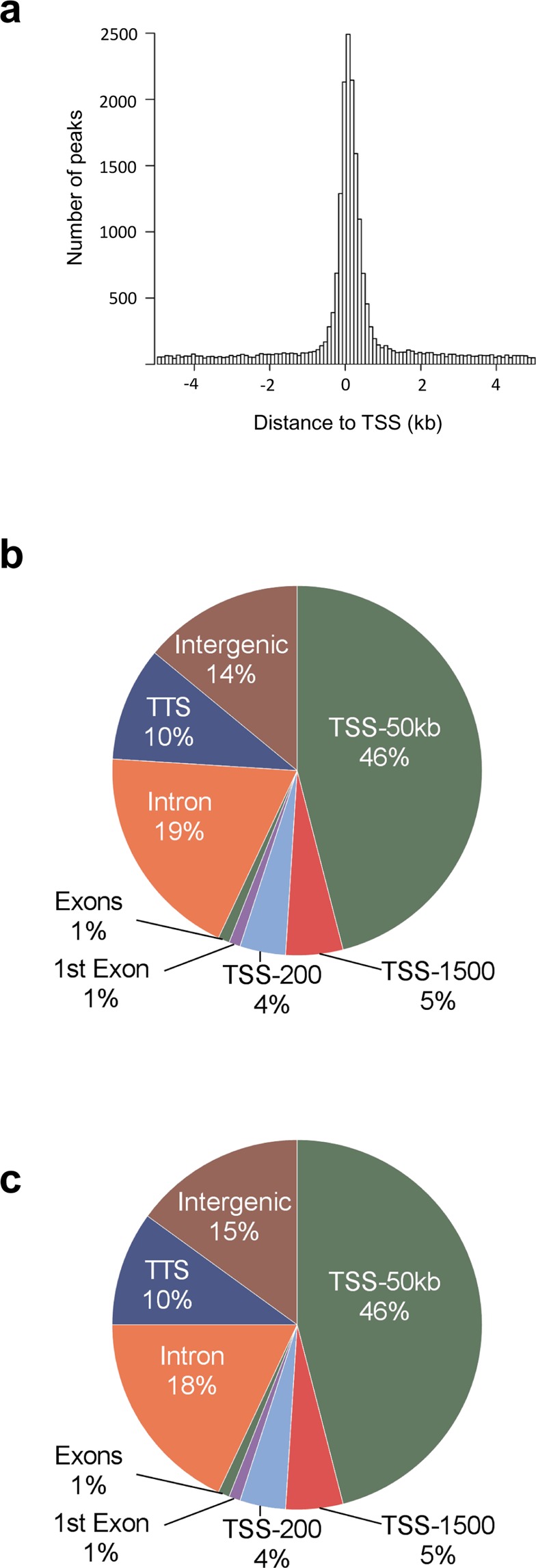
(**a**) Histogram showing the distance from the nearest transcription start site (TSS) for all islet ATAC-seq peaks. **(b-c)**  Proportions of islet ATAC-seq peaks annotated to different genomic regions in (**b**) non-diabetic donors and (**c**) donors with type 2 diabetes. Here, TSS-50 kb represents 1,501–50,000 bp upstream of the TSS, TSS-1500 represents 201–1,500 bp upstream of the TSS, TSS-200 represents 1–200 bp upstream of the TSS, and TTS represents 1–10,000 bp downstream of the transcript termination site.

We further examined the genomic distribution of ATAC-seq open chromatin peaks. As anticipated, a large proportion of ATAC-peaks are located close to TSS (Fig. 2a). Moreover, accessible regions of chromatin show a similar genomic distribution in islets from diabetic and non-diabetic donors (Fig. 2b,c).

We next tested if genetic variants associated with T2D as well as SNPs in linkage disequilibrium (LD) with these overlap with our islet ATAC-seq peaks (Supplemental Table 2). We used 128 SNPs associated with T2D in Scott *et al*.^23^ and 2,207 SNPs in LD with these based on r^2^ = 0.8–1, 1000 genomes, phase 3 and hg19 (http://raggr.usc.edu/). SNPs with a minor allele frequency less than 0.01 were excluded. We found 13 SNPs from Scott *et al*. and 67 LD SNPs which were located within our islet ATAC-seq peaks (Supplemental Table 6). Interestingly, these include SNPs linked to well-studied candidate genes for T2D such as rs7903146, rs2237897, rs757209, rs11708067 and rs878521 which are linked to *TCF7L2*, *KCNQ1*, *HNF1B*, *ADCY5* and *GCK*, respectively. Also the 67 SNPs in LD with known T2D SNPs are linked to key diabetes genes such as *GIPR*, *KCNJ11*, *GLIS3*, *IGF2BP2*, *FTO*, *THADA*, *IRS1* and *PPARG*.

### Enrichment of histone modifications and enhancer elements at open chromatin regions in human islets

**Figure 3:**
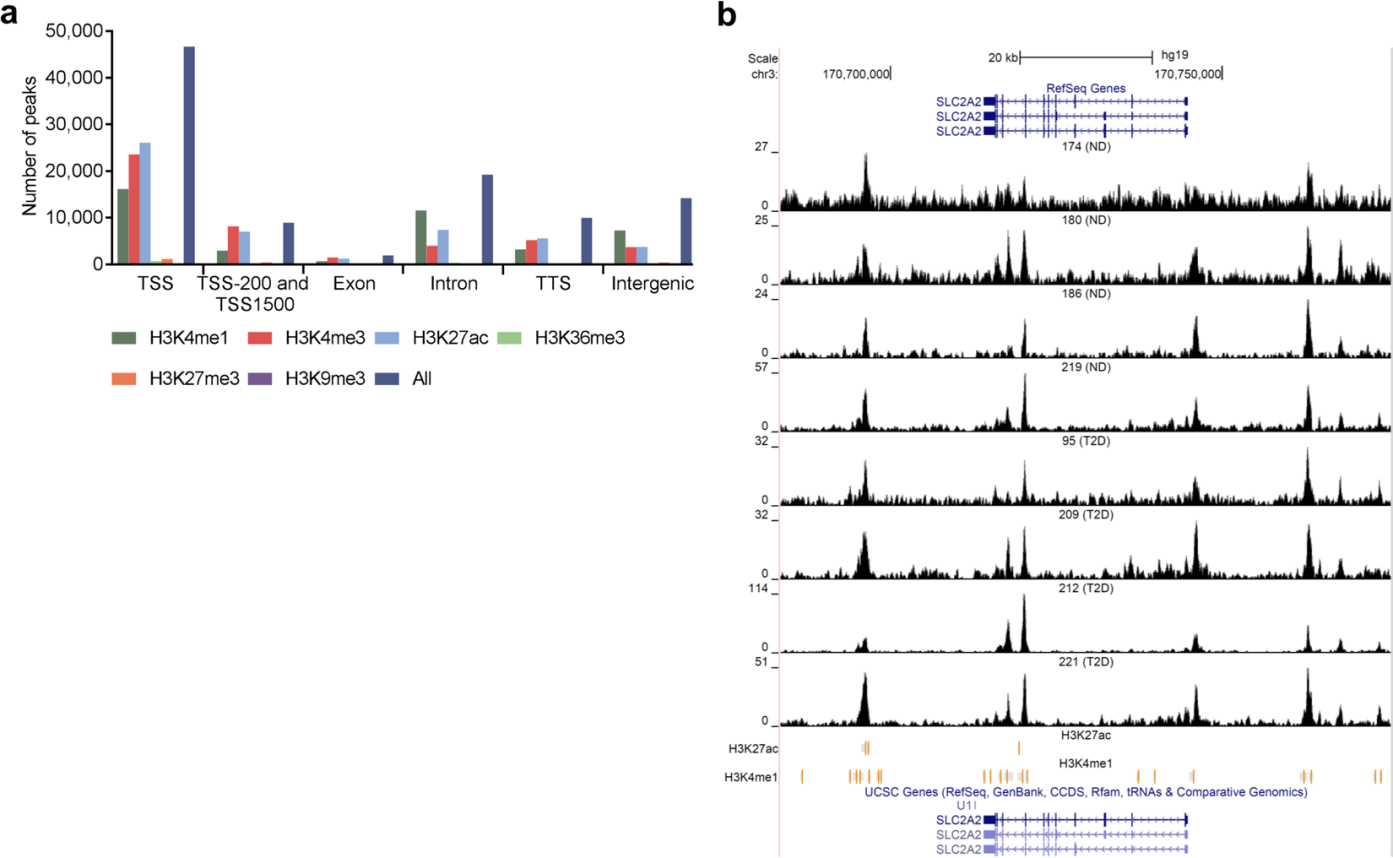
(**a**) Bar graph of overlapping islet ATAC-seq peaks in non-diabetic donors and different histone modifications. Based on chi-square tests and false discovery rate (FDR) analysis (q < 0.001, P < 9 × 10^−153^) more islet ATAC-seq peaks than expected overlapped with H3K4me1, H3K4me3, and H3K27ac and less peaks than expected overlapped with H3K27me3, H3K9me3 and H3K36me3. (**b**) Representative sequencing tracks for the *SLC2A2* locus show ATAC-seq peaks that overlap with H3K4me1 and H3K27ac in human islets. The ATAC-seq data have been normalized to take sequencing depth into account and the scale on the y-axis was chosen for optimal visualization of peaks for each sample.

Post-translational modifications of histones can be used to classify cis-REs such as promoters and enhancers. To further classify the islet ATAC-seq peaks, we intersected them with ChIP-seq data of histone modifications in human islets from the Roadmap Epigenomics Consortium^24^. Histone marks associated with active chromatin (H3K4me1, H3K4me3, and H3K27ac) were enriched at islet ATAC-seq peaks of non-diabetic donors (q < 0.001) (Fig. 3a). In contrast, only a small fraction of histone marks associated with heterochromatin (H3K27me3 and H3K9me3) overlap with ATAC-seq peaks (Fig. 3a). A similar overlap between histone modifications and ATAC-seq peaks was observed in islets from T2D donors (Supplemental Fig. 3).

We also studied enrichment of histone modifications at islet open chromatin regions annotated to different genomic regions i.e. TSS, exons, introns, TTS and intergenic regions (Fig. 3a and Supplemental Fig. 3). For example, the mark associated with active promoters, H3K4me3, is enriched at the open chromatin regions (ATAC-seq peaks) annotated to TSS-200 and TSS-1500 regions (q < 0.001), whereas, the heterochromatin mark H3K27me3 only overlaps with ~1.5% of ATAC-seq peaks in these regions. Other modifications associated with active genes (H3K4me1 and H3K27ac) are also enriched at open chromatin regions annotated to TSS-200 and TSS-1500 regions (q < 0.001).

H3K4me1 and H3K27ac are known to be enriched at enhancer regions. Moreover, the presence of both H3K27ac and H3K4me1 indicates active enhancers while H3K4me1 alone associates with inactive enhancers^25,26^. Open chromatin regions annotated to intron and intergenic regions may also represent enhancers. We found that histone marks associated with enhancer regions are enriched at ATAC-seq peaks annotated to intron and intergenic regions in islets (q < 0.001) (Fig. 3a and Supplemental Fig. 3). We further used this approach to classify active enhancers in the open chromatin regions of islets and identified ~5,000 ATAC-seq peaks at introns and ~2,000 peaks at intergenic regions that overlap with both H3K4me1 and H3K27ac, which is more than expected by chance (q < 0.001) (Fig. 3a, Supplemental Fig. 3 and Supplemental Tables 3–4). Interestingly, some ATAC-seq peaks overlapping with both these marks are annotated to genes that have islet specific function and/or have been associated with diabetes by GWAS e.g., *TCF7L2*, *SLC2A2*, *FOXO1* and *HNF1B*^27^. Sequencing tracks annotated to *SLC2A2* that overlap with both H3K4me1 and H3K27ac are presented in Fig. 3b and Supplemental Fig. 4.

Furthermore, the FANTOM5 project has identified (permissive) functional enhancer candidates across different human cell types and tissues by using cap analysis of gene expression data (CAGE-tag)^28^. We used FANTOM5 data to further classify the islet open chromatin peaks and found 6,475 ATAC-seq peaks that are located at permissive enhancer regions. These 6,475 FANTOM5 enhancer / open chromatin regions overlap with 2,109 regions also covered by H3K4me1-H3K27ac marks (Supplemental Table 7). Numerous genes annotated to these putative enhancer regions e.g. *CACNA1D*, *GLIS3*, *GRB10*, *HDAC9*, *HNF1B*, *INSIG2*, *PAX6*, *PDK4*, *SLC2A2* and *TXNIP* are known to be involved in T2D and islet function^27,29–32^.

**Figure 4:**
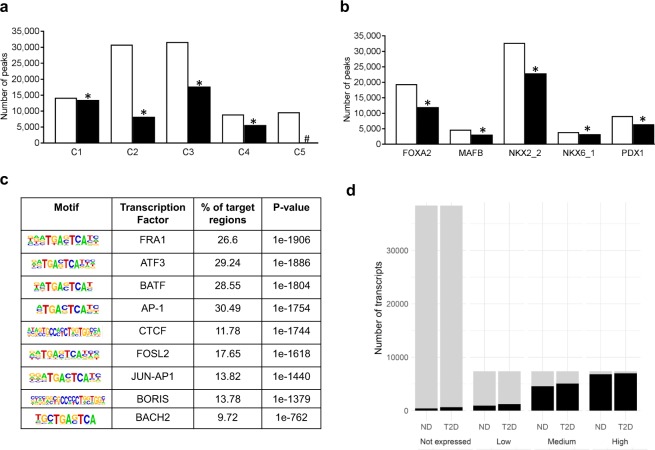
(**a**) Bar graph of overlapping classified promoters (C1 sites), inactive enhancers (C2 sites), active enhancers (C3 sites), CTCF bound sites (C4 sites) and other sites (C5) and islet ATAC-seq peaks generated in non-diabetic donors. White bars (Peaks) represent classified promoter regions generated by Pasquali *et al*.^11^, black bars (Overlaps) represent ATAC-seq peaks that overlap with classified promoter regions. Based on chi-square tests there were significantly more ATAC-seq peaks than expected by chance overlapping with C1, C2, C3 and C4 (*p < 0.00001, q < 0.01) while significantly less than expected overlapping with C5 (#p < 0.00001, q < 0.01). (**b**) Bar graph of overlapping transcription factor binding sites and islet ATAC-seq peaks generated in non-diabetic donors. White bars (All sites) represent transcription factor binding sites classified by Pasquali *et al*.^11^, while black bars (Overlaps) represent ATAC-seq peaks that overlap with transcription factor binding sites. The binding of all these transcription factors was enriched in islet ATAC-seq peaks with p < 0.001 (*q < 0.018). The classified promoters and transcription factor binding sites were generated by Pasquali *et al*.^11^. (**c**) Enrichment of transcription factor recognition sequences in ATAC-seq peaks of non-diabetic donors based on HOMER^17^. (**d**) Islet ATAC-seq peaks are enriched close to high-expressed transcripts. Here, we identified non-expressed transcripts based on transcript per million <0.1 and then divided the expressed transcripts into three equally sized groups that we categorize into low-, medium- and high-expressed transcripts. Proportion of non-, low-, medium-, and high-expressed transcripts that are within 1500 bp of a peak summit. As seen, ATAC-seq peaks were annotated to the majority of high-expressed transcripts, while only a small proportion of non-expressed genes have open chromatin nearby. There was also a clear gradual increase in the proportion of genes with increasing expression level. All three groups of expressed genes were significantly enriched based on chi-square testing (q < 0.001). T2D represents type 2 diabetes and ND represents non-diabetic. Gray region represents genes >1500 bp away from an ATAC-seq peak and black regions represents genes ≤1500 bp away from an ATAC-seq peak.

To further classify the open chromatin regions in human islets, we used data generated by Pasquali *et al*. where promoters, inactive enhancers, active enhancers, and CTCF bound sites were classified^11^. A large number of their classified promoters (C1 sites), active enhancers (C3 sites) and CTCF bound sites (C4 sites) are located at islet ATAC-seq open chromatin regions in non-diabetic donors (Fig. 4a and Supplemental Table 8). However, smaller proportions of inactive enhancers (C2 sites) and other sites (C5 sites) overlap with the islet ATAC-seq open chromatin regions. These data further show that open chromatin regions are mainly accessible to active and functional REs. It should further be noticed that genes of importance for islet function and diabetes such as *PDX1*, *TCF7L2*, *FOXA2*, *FOXO1*, *NEUROD1* and *BACH2* have C1, C3 or both these sites at their ATAC-seq open chromatin regions. Similar results were found in T2D donors, where also large proportions of C1, C3 and C4 sites overlap with the open chromatin regions, while smaller proportions of C2 and C5 sites overlap with ATAC-seq peaks (Supplemental Fig. 5a).

### Enrichment of transcription factor binding at open chromatin regions in human islets

We proceeded to relate the ATAC-seq open chromatin regions with genomic binding of islets-specific TFs using ChIP-seq data from Pasquali *et al*. where they mapped FOXA2, MAFB, PDX1, NKX6.1 and NKX2.2 binding in human islets^11^. The binding of all these TFs was enriched in islet ATAC-seq peaks (p < 0.001) (Fig. 4b and Supplemental Fig. 5b), supporting that most of these TFs are located at chromatin accessible regions. We then used HOMER to identify enriched putative TF motifs in the open chromatin regions of islets (Supplemental Table 9). The most significantly enriched motifs in the open chromatin regions of non-diabetic islet donors include those of FRA1, ATF3, BATF, AP-1, CTCF, FOSL2, AP-1, BORIS, and BACH2 (Fig. 4c). We also observed significant enrichment of motifs for islet specific TFs including MAFA, NEUROD1, PDX1 and NKX6.1 in the ATAC-seq peaks (Supplemental Table 9).

### Expression levels in relation to open chromatin regions in human islets

To explore the relationship between open chromatin and gene expression levels, we used islet ATAC-seq and RNA-seq data generated in the same donors (5 T2D and 5 non-diabetic). A total of 60,517 GENCODE transcripts were categorized as either not expressed (38,428 transcripts, based on Transcript per Million (TPM) < 0.1) or as low- (7,363), medium- (7,363) and high-expressed (7,363) transcripts based on three equally sized groups of expressed transcripts (TPM > 0.1). While ATAC-seq peaks were annotated to the majority of high-expressed transcripts, only a small proportion of non-expressed genes have open chromatin nearby (Fig. 4d). There was also a clear gradual increase in the proportion of genes with annotated ATAC-seq peaks from the low- to medium- and high-expression genes. Notably, several genes with important function in islets such as *PDX1*, *GCG*, *FOXA2* and *NEUROD1* are in the high-expressed category (data not shown). These data support that the chromatin is more open in high-expressed than in non-expressed genes. We also generated a Venn diagram showing that ATAC-seq peaks were annotated to the majority of high-expressed transcripts, while only a small proportion of non-expressed genes have open chromatin nearby (Supplemental Fig. 6).

### Differential methylated regions at open chromatin regions in human islets

DNA methylation may inhibit the binding of TFs to DNA in a cell specific manner. We recently identified ~25,000 differentially methylated regions (DMRs) in islets of T2D versus non-diabetic donors by using whole-genome bisulfite sequencing and these DMRs were enriched in binding sites for islet-specific TFs^7^. Here, we found that these T2D-associated DMRs are located at 7,412 open chromatin regions in human islets, including regions annotated to *PDX1* and *SLC20A2* (Supplemental Table 10).

### Open chromatin regions differ in islets from T2D versus non-diabetic donors

**Figure 5:**
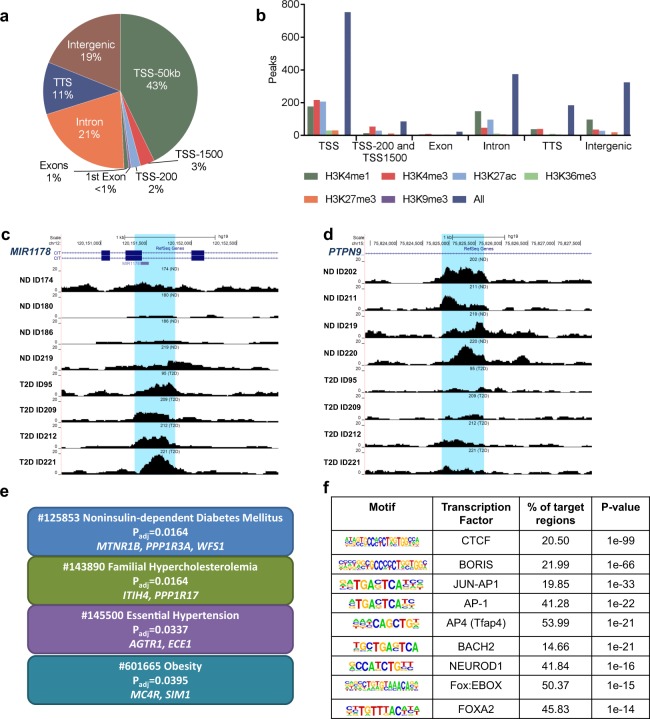
(**a**) Proportions of differential islet ATAC-seq peaks of type 2 diabetic versus non-diabetic donors annotated to different genomic regions. (**b**) Bar graph of differential islet ATAC-seq peaks of type 2 diabetic versus non-diabetic donors that overlap with histone modifications. (**c-d**) Sequencing tracks of ATAC-seq peaks that are more prevalent in donors with type 2 diabetes (annotated to *MIR1178*) (**c**) or non-diabetics (annotated to *PTPN9*) (**d**). The ATAC-seq data have been normalized to take sequencing depth into account. (**e**) Disease related pathways based on enrichment of genes annotated to differential islet ATAC-seq peaks of type 2 diabetic versus non-diabetic donors. The same pathways were significant when analyzing genes annotated to the 1,044 peaks enriched in T2D versus non-diabetic donors (P_adj_ = 0.0159, P_adj_ = 0.0175, P_adj_ = 0.324 and P_adj_ = 0.0425, respectively). P_adj_ are adjusted by FDR. (**f**) Enrichment of transcription factor recognition sequences in differential islet ATAC-seq peaks of type 2 diabetic versus non-diabetic donors based on HOMER^17^.

To identify T2D-associated changes in the open chromatin landscape, we used Fisher’s exact test to examine whether islet ATAC-seq peaks were more prevalent in T2D versus non-diabetic donors. Here, 1,078 open chromatin peaks were found to differ between T2D and non-diabetic islet donors (Supplemental Table 11). The majority (1,044) of these 1,078 peaks were enriched in T2D donors. The genomic distribution of these 1,078 peaks is presented in Fig. 5a and some of them are marked with histone modifications associated with open chromatin (Fig. 5b). Interestingly, several of these genes e.g. *CLEC16A*, *ELAVL4* (also known as *HuD*), *FOXO3*, *FST*, *GLIS3*, *MTNR1B*, *PARK2*, *WFS1* and *ZMIZ1* have previously been associated with T2D by GWAS and/or found to affect islet function^7,33–40^. Sequencing tracks of ATAC-seq peaks that are more prevalent in either donors with T2D (e.g. *MIR1178*) or non-diabetics (e.g *PTPN9*) are presented in Fig. 5c,d and Supplemental Fig. 7a,b. Interestingly, MIR1178 is a suppressor of CHIP, also known as STUB1 (STIP1 homology and U-box containing protein 1), and loss of CHIP is associated with diabetes^41,42^.

We then used WebGestalt (http://www.webgestalt.org) and the functional database OMIM to identify disease-related pathways with enrichment of genes that had islet ATAC-seq peaks with a different prevalence in T2D versus non-diabetic donors. Interestingly, categories including Noninsulin-dependent Diabetes Mellitus, Familial Hypercholesterolemia, Essential Hypertension and Obesity were significantly enriched (Fig. 5e). Of note, Familial hypercholesterolemia, hypertension and obesity are linked to risk of T2D and/or islet function^36,43,44^. The same pathways were significant when only analyzing genes annotated to peaks that are enriched in T2D donors, while no pathway was significant when analyzing genes annotated to peaks that are enriched in non-diabetic donors.

We proceeded to examine if ATAC-seq peaks with a different prevalence in T2D islets are located in specific REs by using data from Pasquali *et al*.^11^. Among these 1,078 open chromatin peaks, 3.7% overlap with classified promoters (C1 sites), 19.4% with inactive enhancers (C2 sites), 22.4% with active enhancers (C3 sites) and 11.7% with CTCF bound sites (C4 sites) (Supplemental Table 12). Hence, the peaks with different prevalence in T2D islets are most common at classified enhancer regions (~42%).

To identify TF motifs significantly enriched at the 1,078 ATAC-seq peaks with different prevalence in T2D, we performed a motif search using HOMER. Intriguingly, motifs specific to key TFs in islets, including BORIS, BACH2, FOXO1, FOXA2, NEUROD1, MAFA and PDX1 as well as the motif for CTCF were significantly enriched in the ATAC-seq peaks with different prevalence in T2D islets (Supplemental Table 13). Some of the most significant motifs are presented in Fig. 5f. CTCF is an insulator transcription factor, which plays a key role in maintaining higher order chromatin structure by facilitating the interactions between the regulatory sequences. We further examined if the expression of any of the TFs presented in Fig. 5f exhibit differential expression in islets from donors with T2D. Interestingly, the expression of *BACH2* was elevated in T2D versus control human islets (p < 0.036)^5^.

We then determined if the 128 SNPs associated with T2D^23^ and 2,207 SNPs in LD with these are located within the 1,078 islet ATAC-seq peaks with different prevalence in T2D islets. We found that rs3821943 and rs508419 annotated to *WFS1* and *ANK1*, respectively, are located in ATAC-seq peaks with different prevalence in T2D islets (Supplemental Tables 6 and 11).

We next examined whether the genes annotated to ATAC-seq peaks with different prevalence in T2D islets also showed altered expression in T2D versus non-diabetic islets. Using the RNA-seq data from a previous study^5^, we identified 90 genes that have both differential gene expression and different open chromatin regions in T2D islets (Supplemental Table 14). These include genes with known function in islets and diabetes such as *ATP6V1H*, *SOX6*, *SOCS1* and *STX11*^45–48^. ATAC-seq peaks annotated to *SOX6* are presented in Supplemental Fig. 8a. Moreover, based on RNA-seq data from the donors included the present study, we found 54 genes that had both differential gene expression and open chromatin regions in T2D islets (Supplemental Table 15). The majority of these genes (~70%) had higher expression and more open chromatin in T2D islets.

We then used R Diffbind and edgeR packages^19^. These data are presented in a volcano plot in Supplemental Fig. 8b. We found 88 ATAC-seq peaks that were both differential in T2D islets and more prevalent in T2D versus non-diabetic donors based on Fisher’s exact test (Supplemental Table 16). These include *MIR1178* (Fig. 5c), *BACH1*, *GABRA2* and *STX11* which are known to play a role in beta-cells, islets and diabetes^41,42,49^. Using gene ontology, we further found that the anion transmembrane transporter activity pathway (http://amigo.geneontology.org/amigo/term/GO:0008509) was significantly enriched among the genes annotated to the 88 differential ATAC-seq peaks (P = 8 × 10^−6^, q = 0.02, *CTNS*, S*LC16A14*, *ANO6*, *ANO5*, *GABRA2* and *SLC16A7* contributed to the enrichment).

**Figure 6:**
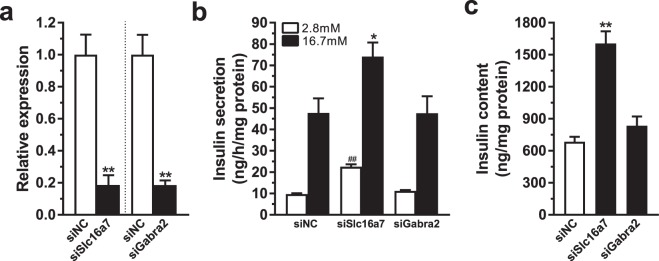
(**a**) Knockdown of *Slc16a7* and *Gabra2* as quantified by qPCR (**p < 0.01). (**b**) Knockdown of *Slc16a7* increased both basal (2.8 mM) and glucose-stimulated (16.7 mM) insulin secretion, while knockdown of *Gabra2* has no significant effect (^##^p < 0.01 vs siNC 2.8 mM, *p < 0.05 vs siNC 16.7 mM). (**c**) Insulin content is increased in cells deficient for *Slc16a7* while there is no significant difference after knockdown of *Gabra2* (**p < 0.01).

We finally asked if genes annotated to ATAC-seq peaks with different prevalence (based on both Fisher’s exact test and R Diffbind) and with differential expression in T2D versus non-diabetic islets have a functional role in beta-cells. Two genes, *SLC16A7* and *GABRA2*, were selected for functional follow-up experiments based on these criteria (Supplemental Tables 14 and 15) together with a potential role in beta-cell function^50,51^. Of note, both *GABRA2* and *SLC16A7* contributed to the enrichment of the Anion transmembrane transporter activity pathway which was significantly enriched among the genes annotated to the 88 differential ATAC-seq peaks. While *SLC16A7* showed increased expression and enrichment of ATAC-seq peaks, *GABRA2* had decreased expression and less ATAC-seq peaks in T2D islets. First, Slc16a7 and Gabra2 were silenced in rat clonal beta-cells (Fig. 6a) and we then measured insulin secretion at basal (2.8 mM) and stimulatory (16.7 mM) glucose levels. Silencing of Slc16a7 resulted in increased glucose-stimulated and basal insulin secretion as well as increased insulin content (Fig. 6b,c), which fits with the fact that non-diabetic people have lower expression levels and fewer ATAC-seq peaks of this gene. On the other hand, silencing of Gabra2 only had a nominal effect on basal insulin secretion.

## Discussion

This study used ATAC-seq to establish T2D-associated changes in the open chromatin landscape of human islets. Interestingly, we found 1,078 differential ATAC-seq peaks in T2D versus control islets. T2D is driven by interactions between genetic and environmental factors. A major breakthrough in genetic research on T2D came with GWAS where ~100 loci were identified^2^. GWAS placed the pancreatic islets in the center of attention as many of the loci were associated with impaired insulin secretion^3^. Although large efforts have been made to understand how these SNPs may cause disease further studies are needed. Interestingly, several risk genes identified by GWAS such as *HHEX*, *HMGA2*, *GLIS3*, *MTNR1B* and *WFS1*^35,36,38,52^ had differential ATAC-seq peaks in T2D islets annotated to them. It is possible that T2D-associated SNPs change the chromatin structure and thereby gene regulation. In support for this, we have previously shown that SNPs linked to *HHEX*, *HMGA2* and *WFS1* associate with epigenetic changes, e.g. differential DNA methylation, in human islets^6,38^. Here, we found T2D-associated differences in ATAC-seq peaks annotated to genes which also show differential DNA methylation patterns in islets from T2D versus non-diabetic donors e.g. *SOX6*^6,7^ (Supplemental Fig. 8c). Additionally, several of the genes annotated to differential ATAC-seq peaks have previously been shown to affect insulin secretion. For example, *MTNR1B* expression is increased and insulin secretion decreased in islets from G-allele carriers which increases T2D risk. MTNR1B deficient mice secreted more insulin and MTNR1B overexpression affect insulin secretion in beta-cells^35^. Moreover, beta-cell mass and insulin secretion were reduced in rats where exon 5 of *Wfs1* was deleted^53^. Also, GLIS3 controlled beta-cell proliferation in response to high-fat feeding and Glis3 deficiency leads to diabetes^54^. The potential causal consequences of differences in ATAC-seq peaks between donors with T2D and controls should be examined further and some caution should be considered regarding the gene annotations of these peaks. Here, we performed functional follow-up experiments of two identified genes with both differential ATAC-seq peaks and expression in islets from donors with T2D versus non-diabetic controls. We found that knockdown of *Slc16a7*, which exhibits fewer ATAC-seq peaks and lower expression in islets from non-diabetic donors, increased both insulin secretion and content in beta-cells. This supports a role for the identified chromatin differences in the secretory insufficiency that is characteristic of T2D. We cannot exclude that altered levels of Gabra2 affect other phenotypes than impaired insulin secretion, characterizing islets from donors with T2D.

Insufficient insulin secretion is a hallmark of T2D. Altered expression of insulin and numerous other genes seems to contribute to islet-dysfunction^5,55^. Gene expression is regulated by TFs that bind to DNA in open chromatin regions in a sequence-specific manner^9,10,56^. Given this, we studied TF binding and putative TF binding motifs in open chromatin regions of T2D and non-diabetic islets. As may be expected, ATAC-seq peaks were enriched in regions occupied by islet-specific TFs such as FOXA2, MAFB, NKX2.2, NKX6.1 and PDX1. These TFs are important for islet development and function as well as for proper insulin expression^21^. For example, PDX1 regulates development of beta-cells and insulin expression in mature beta-cells. While expression of PDX1 seems to be regulated by epigenetic mechanisms in T2D islets^6,7,57^, its function may be controlled by the chromatin structure and PDX1’s ability to bind to its target genes. Putative TF binding motifs were also enriched in the islet ATAC-seq peaks. Interestingly, the FRA1 motif was the most significantly enriched among islet ATAC-seq peaks. Our result is in line with data from a recent study where the motif for FRA1 was enriched in ATAC-seq peaks in alpha- and beta-cells^14^. FRA1 is involved in MAPK signaling and oxidative stress pathways. However, there is limited knowledge of its role in islets and future studies evaluating its binding and function would be interesting. The motif for CTCF was also significantly enriched in our islet ATAC-seq peaks as well as in the peaks that differed between T2D and non-diabetic islet donors. Varshney *et al*. previously did a reconstruction of the CTCF motif using ATAC-seq TF footprint allelic bias data of two islet donors^15^ and Ackerman *et al*. found this motif to be enriched in ATAC-seq peaks from sorted alpha-cells^14^. Moreover, alpha- and beta-cell-specific peaks e.g. *ARX*, *GCG*, *DPP4* and *SLC27A6* presented by Ackerman *et al*. were also identified in our islet ATAC-seq data^14^.

Epigenetic modifications are closely linked to chromatin structure. We have studied epigenetic alterations in T2D versus control islets by genome-wide DNA methylation analyses^6,7^. Here, we integrated ATAC-seq peaks with the methylome and found that T2D-associated DMRs are located at 7,412 open chromatin regions in human islets. Earlier, we found DMRs in and decreased expression of *PARK2* in T2D versus control islets^7^. PARK2 regulates mitochondrial function in beta-cells and its silencing impaired insulin secretion. Here, we fund differential ATAC-seq peaks annotated to *PARK2* in T2D islets, further supporting its role in diabetes. Given the importance of posttranslational modifications of histones, we related islet ATAC-seq peaks to histone marks associated with enhancers, active promoters and inactive genes. While histone marks of active enhancers and promoters were enriched, histone marks of inactive genes were underrepresented in open chromatin regions. These results were further supported by CAGE data and data generated by Pasquali *et al*.^11,28^ and are in line with muscle ATAC-seq data^58^.

There are advantages with ATAC-seq compared with earlier methods. It requires smaller number of cells and has higher sensitivity and resolution. Indeed, we only used ~30 islets per donor^9,12^. It should be noted that the number of islets used for ATAC-seq was optimized and 25–30 islets per donor gave the best result. Importantly, our data seem technically sound since a large proportion of our ATAC-seq open chromatin peaks overlapped with ENCODE validated peaks. Of note, islets contain several cell types. However, our previous data support that there are no differences in islet cell composition between T2D and non-diabetic donors^6^. Moreover, in the present study, we found no differences in expression of islet cell specific genes in T2D versus control donors further supporting no differences in islet cell composition in our cohort (Supplemental Fig. 9).

Additionally, since the current study included data from 15 donors, the differences in islet ATAC-seq peaks identified between donors with T2D and controls should be replicated in future studies. The donors with T2D have a higher BMI than the controls and it is possible that this affects our results. Subsequently, it will be of interest to only study the effect of BMI on the chromatin structure in human islets. Also since whole islets contain several different cell types, and these cell types may be in different proportions in different donors, future studies should perform ATAC-seq in sorted islet cells from donors with T2D and controls. To maximize our statistical power regarding analysis of RNA-seq data, we used both published expression data from a larger number of islet donors including 12 with T2D and 66 controls^5^ as well as RNA-seq data from the donors with available ATAC-seq data.

Together, we provide the first map of open chromatin regions in islets from numerous T2D donors. Dissection of open chromatin regions revealed enrichment for islet-specific TF binding motifs and enhancer regions. Numerous SNPs associated with T2D reside within islet ATAC-seq peaks. T2D candidate genes showed differential ATAC-seq peaks in diabetic versus non-diabetic islets. Our study improves current understanding of the link between altered chromatin structure and disease.

## Supplementary information

Supplementary info

Table S2

Table S3

Table S4

Table S5

Table S6

Table S7

Table S8

Table S9

Table S10

Table S11

Table S12

Table S13

Table S14

Table S15

Table S16

## Data Availability

Datasets supporting the conclusions of this article are available in the GEO repository, accession number GSE50398 and GSE129383, or are available upon request.
