# Author Correction: ATAC-seq reveals alterations in open chromatin in pancreatic islets from subjects with type 2 diabetes

**DOI:** 10.1038/s41598-020-58485-7

**Published:** 2020-01-29

**Authors:** Madhusudhan Bysani, Rasmus Agren, Cajsa Davegårdh, Petr Volkov, Tina Rönn, Per Unneberg, Karl Bacos, Charlotte Ling

**Affiliations:** 1 Epigenetics and Diabetes Unit, Department of Clinical Sciences, Lund University Diabetes Centre, Lund University, Scania University Hospital, Malmö, Sweden; 2 Department of Biology and Biological Engineering, National Bioinformatics Infrastructure Sweden, Science for Life Laboratory, Chalmers University of Technology, Göteborg, Sweden; 3 Department of Cell and Molecular Biology, National Bioinformatics Infrastructure Sweden, Science for Life Laboratory, Uppsala University, Uppsala, Sweden

Correction to: *Scientific Reports* 10.1038/s41598-019-44076-8, published online 23 May 2019

Fifteen supplementary datasets containing ATAC-seq data were omitted from the original version of this Article. This has been corrected in the HTML version of the Article; the PDF version was correct at time of publication.

