# Supplementary material for: ATAC-seq reveals alterations in open chromatin in pancreatic islets from subjects with type 2 diabetes
Source: Sci Rep. 2019 May 23;9:7785. doi: 10.1038/s41598-019-44076-8 (PMC6533306; doi:10.1038/s41598-019-44076-8)
Supplement: Supplementary file 1 — Supplementary info [file 41598_2019_44076_MOESM1_ESM.pdf]

Supplemental Material and Methods

ATAC-seq reveals alterations in open chromatin in pancreatic islets from subjects with type 2 diabetes

Madhusudhan Bysani, Rasmus Agren, Cajsa Davegård, Petr Volkov, Tina Rönn, Per Unneberg, Karl Bacos and Charlotte Ling

ATAC-seq

Briefly, islets were washed twice with ice cold 1xPBS by spinning at 500g for 5mins at 4°C. Cell lysis was performed with ATAC lysis buffer (10mM Tris-HCl, pH 7.4, 3mM $MgCl_2$, 10mM NaCl and 0.1% IGEPAL CA-630) by incubating on ice for 10mins and nuclei were then collected by spinning at 500g for 10mins at 4°C. The transposition reaction mix (12.5µl of 2X TD buffer, 2µl of Tn5 transposase (Illumina, San Diego, CA, USA) and 10.5µl of nuclease free water) was added to nuclei and incubated at 37°C for 1hour before the reaction was cleaned using MinElute cleanup kit (Qiagen, Hilden, Germany). Next, 10-12cycles of PCR were performed with transposed DNA using the Illumina Nextera dual index primers. The PCR product was cleaned using PCR purification kit (Qiagen). Resulting libraries were size selected on 1% TAE gel to exclude fragments larger than 800bp. The library quality and fragment size distribution was analyzed by DNA tape station kit (Agilent technologies, Santa Clara, CA, USA). Libraries were quantified using Qubit fluorometer (LifeTechnologies, Paisley, UK) before they were sequenced on Illumina HiSeq2500 platform with 125bp paired-end sequencing. ATAC-seq libraries were sequenced to an average of 101.3 million reads per islet sample, resulting in a total of 1,519 million sequencing reads.

Analysis of ATAC-seq data

Adapter sequences were trimmed from the raw ATAC-seq reads with CutAdapt v1.12[1]. Trimmed reads of each sample were mapped to the reference human genome build GRCh37/hg19 by using Bowtie2 v2.3.2 with default settings[2]. Picard tools

(http://broadinstitute.github.io/picard/) v1.126 was used to remove PCR duplicated reads and for insert size distribution analysis. Reads mapping to mitochondrial DNA and the Y chromosome were filtered and only uniquely mapping paired reads with a quality score≥30 were kept for subsequent analysis. The read start sites were adjusted to represent the center of the transposon binding event (5bp on forward strand and 4bp on reverse strand) as described[3]. Phantompeakqualtools v2.0 was used to calculate the strand cross-correlation and deepTools v2.0.1 was used to calculate correlation between samples[4,5]. MACS2 v2.1.0 was used for peak calling with parameters "--nomodel--nolambda-keep-dup all--call-summits-g hs-B", and peaks were filtered based on default settings and false discovery rate (FDR) < 0.1[6]. Peaks in regions known to show artificially high signal were filtered using the hg19 blacklist file from ENCODE. BEDTools v2.26.0[7] was used for various analyses based on peak locations. Peaks located less than 50bp apart were merged in order to facilitate comparison between samples. At this stage, ~40% of peaks were only present in one sample and these reflect individual-specific peaks. A peak was only considered for subsequent analyses if it was identified in three or more islet donors.

Fisher's exact test was used for an occupancy-based analysis (i.e. to identify islet ATAC-seq peaks that were found in significantly more non-diabetic compared to T2D donors or vice versa). The R Diffbind package and edgeR package[8] were used for an affinity-based analysis (i.e. to identify islet ATAC-seq peaks where the mean number of mapped reads differ between the groups). Gender and sample treatment (frozen/non-frozen) were included as covariates in the design matrix. The whole ATAC-seq workflow was implemented in Snakemake. All sequencing track figures were generated with the UCSC Genome Browser.

Islet RNA-seq

Total RNA was extracted using the AllPrep DNA/RNA mini kit (Qiagen). RNA quality was measured by 2100 Bioanalyzer and 2200 Tape station (Agilent Technologies). RNA quantity was measured by NanoDrop 1000 (NanoDrop Technologies) or Qubit 2.0 Fluorometer (Life Technologies). 1µg of total RNA with a RIN value≥8 was used for library preparation with TruSeq RNA sample preparation kit (Illumina) before sequencing on Illumina HiSeq2000 platform as described[9,10].

Expression values are presented as log2 transcripts per million (TPM) after quality control, normalization and transformation as described[9]. Transcripts were categorized into four categories; transcripts with TPM<0.1 were considered as non-expressed and the remaining transcripts were equally categorized as low-, medium- and high-expressed based on their TPM value[11]. For the purpose of correlating ATAC-seq peaks with mRNA expression, genes were classified based on whether any of their annotated features (TSS, TTS, intron, exon, 3'UTR, 5'UTR) were within 1500 bp of a peak summit.

Overlapping ATAC-seq peaks with public ChIP-seq datasets

Easeq version 1.03[12] was used for co-localization analysis e.g. overlapping ATAC-seq peaks with histone modifications from ENCODE and TF binding[13]. Chi-square tests were used to analyze enrichment of ChIP-seq peaks overlapping with ATAC-seq peaks. Expected frequencies were set to 0.05 of the total number of ChIP-seq peaks found for each studied attribute.

Availability of data

Datasets supporting the conclusions of this article are available in the GEO repository, accession number GSE50398 and GSE129383 or are available upon request.

References

1 Martin, M. Cutadapt removes adapter sequences from high-throughput sequencing reads. . *EMBnet.journal* **17**, 10-12 (2011).

2 Langmead, B. & Salzberg, S. L. Fast gapped-read alignment with Bowtie 2. *Nat Methods* **9**, 357-359, doi:10.1038/nmeth.1923 (2012).

3 Buenrostro, J. D., Giresi, P. G., Zaba, L. C., Chang, H. Y. & Greenleaf, W. J. Transposition of native chromatin for fast and sensitive epigenomic profiling of open chromatin, DNA-binding proteins and nucleosome position. *Nat Methods* **10**, 1213-1218, doi:10.1038/nmeth.2688 (2013).

4 Landt, S. G. *et al.* ChIP-seq guidelines and practices of the ENCODE and modENCODE consortia. *Genome Res* **22**, 1813-1831, doi:10.1101/gr.136184.111 (2012).

5 Ramirez, F. *et al.* deepTools2: a next generation web server for deep-sequencing data analysis. *Nucleic Acids Res* **44**, W160-165, doi:10.1093/nar/gkw257 (2016).

6 Zhang, Y. *et al.* Model-based analysis of ChIP-Seq (MACS). *Genome Biol* **9**, R137, doi:10.1186/gb-2008-9-9-r137 (2008).

7 Quinlan, A. R. & Hall, I. M. BEDTools: a flexible suite of utilities for comparing genomic features. *Bioinformatics* **26**, 841-842, doi:10.1093/bioinformatics/btq033 (2010).

8 Robinson, M. D., McCarthy, D. J. & Smyth, G. K. edgeR: a Bioconductor package for differential expression analysis of digital gene expression data. *Bioinformatics* **26**, 139-140, doi:10.1093/bioinformatics/btp616 (2010).

9 Fadista, J. *et al.* Global genomic and transcriptomic analysis of human pancreatic islets reveals novel genes influencing glucose metabolism. *Proceedings of the National Academy of Sciences of the United States of America* **111**, 13924-13929, doi:10.1073/pnas.1402665111 (2014).

10 Gandasi, N. R. *et al.* Glucose-Dependent Granule Docking Limits Insulin Secretion and Is Decreased in Human Type 2 Diabetes. *Cell Metab* **27**, 470-478 e474, doi:10.1016/j.cmet.2017.12.017 (2018).

11 Volkov, P. *et al.* Whole-Genome Bisulfite Sequencing of Human Pancreatic Islets Reveals Novel Differentially Methylated Regions in Type 2 Diabetes Pathogenesis. *Diabetes* **66**, 1074-1085, doi:10.2337/db16-0996 (2017).

12 Lerdrup, M., Johansen, J. V., Agrawal-Singh, S. & Hansen, K. An interactive environment for agile analysis and visualization of ChIP-sequencing data. *Nat Struct Mol Biol* **23**, 349-357, doi:10.1038/nsmb.3180 (2016).

13 Pasquali, L. *et al.* Pancreatic islet enhancer clusters enriched in type 2 diabetes risk-associated variants. *Nat Genet* **46**, 136-143, doi:10.1038/ng.2870 (2014).

Supplemental Table 1: Strand cross-correlation statistics from phantompeakqualtools. Values of the quality metrics recommended by ENCODE as successful ChIP-seq experiment are NSC > 1.05 and RSC > 0.8. Datasets with NSC values much less than 1.1 (< 1.05) tend to have low signal to noise or few peaks. Datasets with RSC values significantly lower than 1 (< 0.8) tend to have low signal to noise. Quality tag is based on thresholded RSC (codes: -2:Very low, -1:Low, 0:Medium, 1:High, 2:Very high).

Sample	Number of reads	NSC	RSC	Quality tag	Islet preparation
213	69,840,168	1.073482	1.155494	1	Fresh, picked
222	68,981,634	1.103705	1.648971	2	Fresh, picked
95	74,544,246	1.088201	1.024797	1	Frozen, biobank
202	67,713,672	1.325657	1.642671	2	Frozen, picked
180	69720374	1.103942	1.176681	1	Frozen, biobank
168	87157954	1.095505	1.13159	1	Frozen, biobank
221	67281118	1.250516	1.256824	1	Fresh, picked
211	55570840	1.569525	1.153834	1	Frozen, picked
210	67529292	1.215765	1.34549	1	Frozen, picked
186	19196372	1.240378	1.616308	2	Frozen, biobank
209	56758454	1.133337	1.495381	1	Frozen, picked
219	77876938	1.103804	1.51757	2	Fresh, picked
212	52869938	1.542672	1.247734	1	Frozen, picked
174	109492488	1.05789	1.133238	1	Frozen, biobank
220	69596798	1.273368	1.330062	1	Fresh, picked

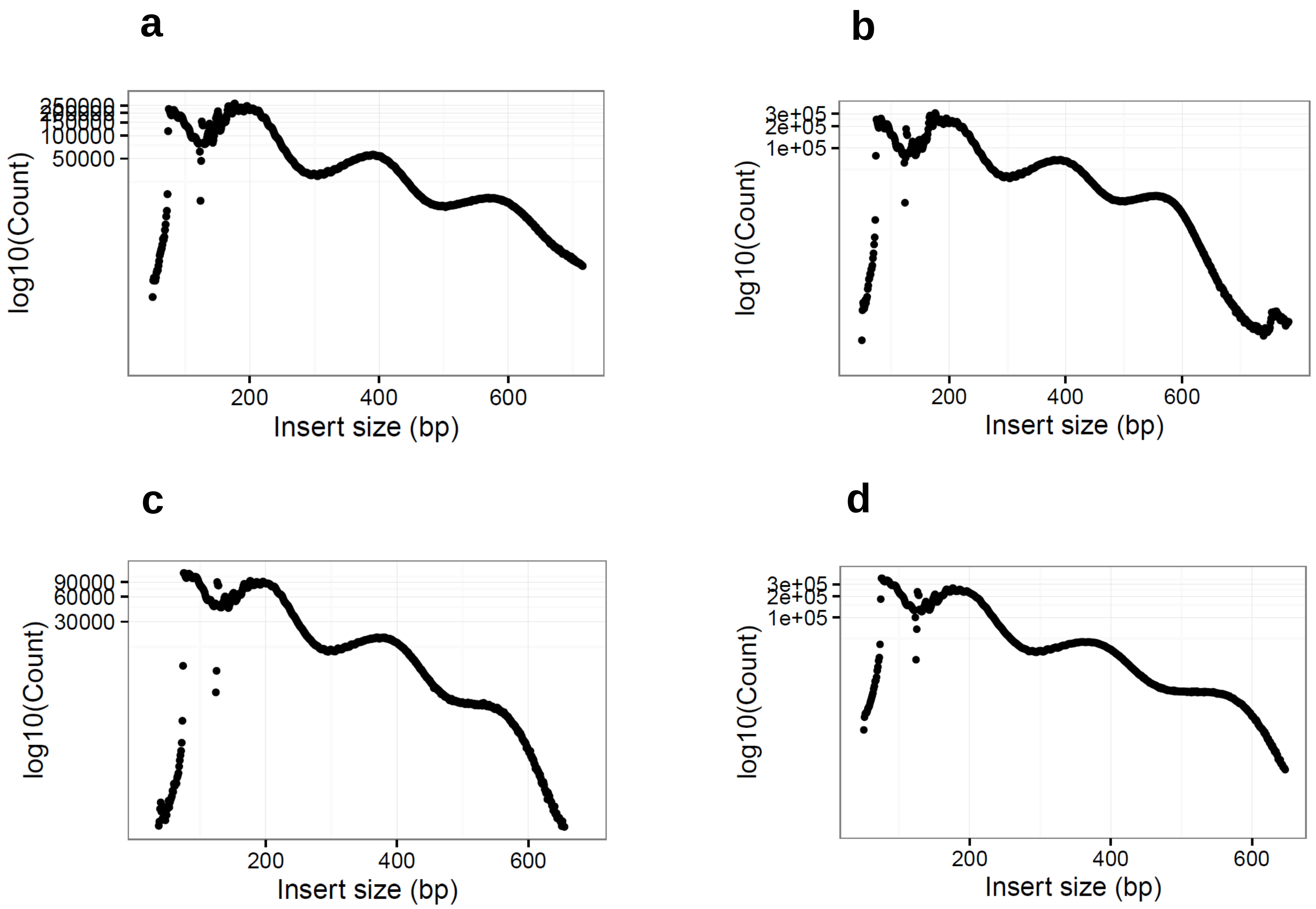

Supplemental Fig. 1 Insert size distributions of islet ATAC-seq data showing clear nucleosome phasing for both **a-b** frozen and **c-d** fresh islets.

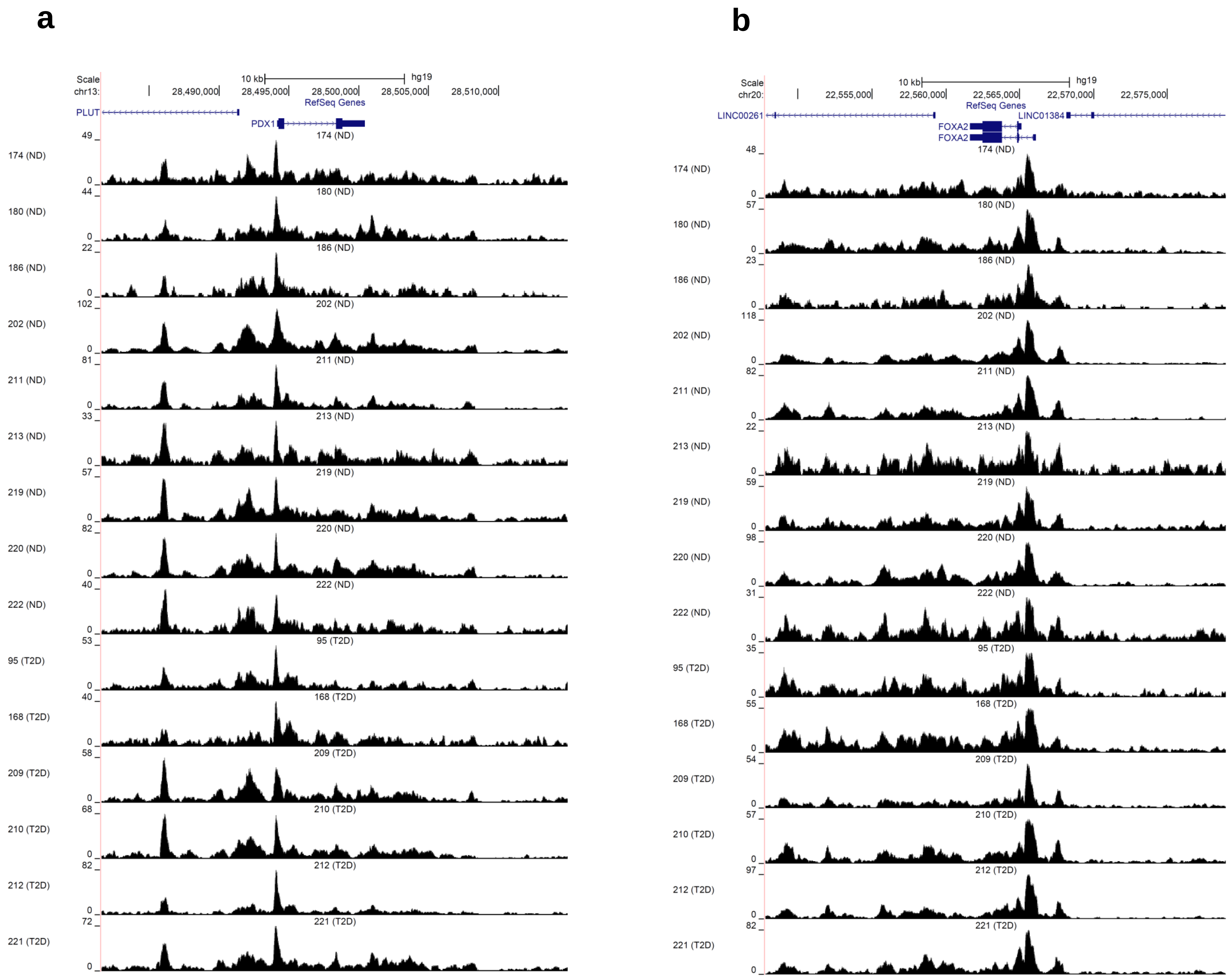

Supplemental Fig. 2 Representative sequencing tracks for **a**) the *PDX1* and **b**) the *FOXA2* locus show distinct ATAC-seq peaks at the promoter and the known enhancer in human islets for all analysed islet donors.

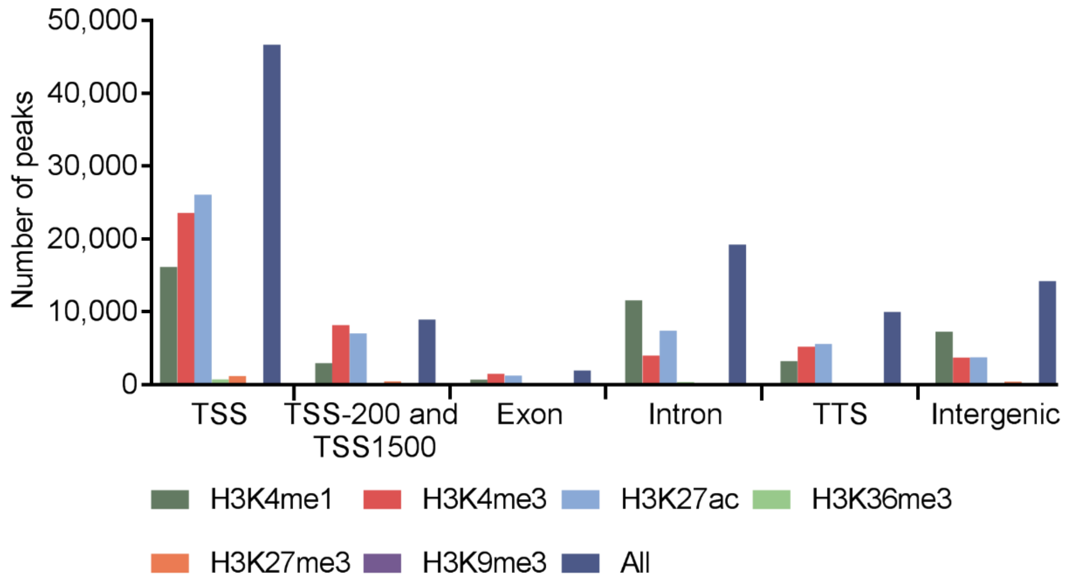

Supplemental Fig. 3 Bar graph of overlapping islet ATAC-seq peaks in donors with type 2 diabetes and different histone modifications. Based on chi-square tests and false discovery rate analysis (q<0.001, P<9x10^-153) more islet ATAC-seq peaks than expected overlapped with H3K4me1, H3K4me3, and H3K27ac and less peaks than expected overlapped with H3K27me3, H3K9me3 and H3K36me3.

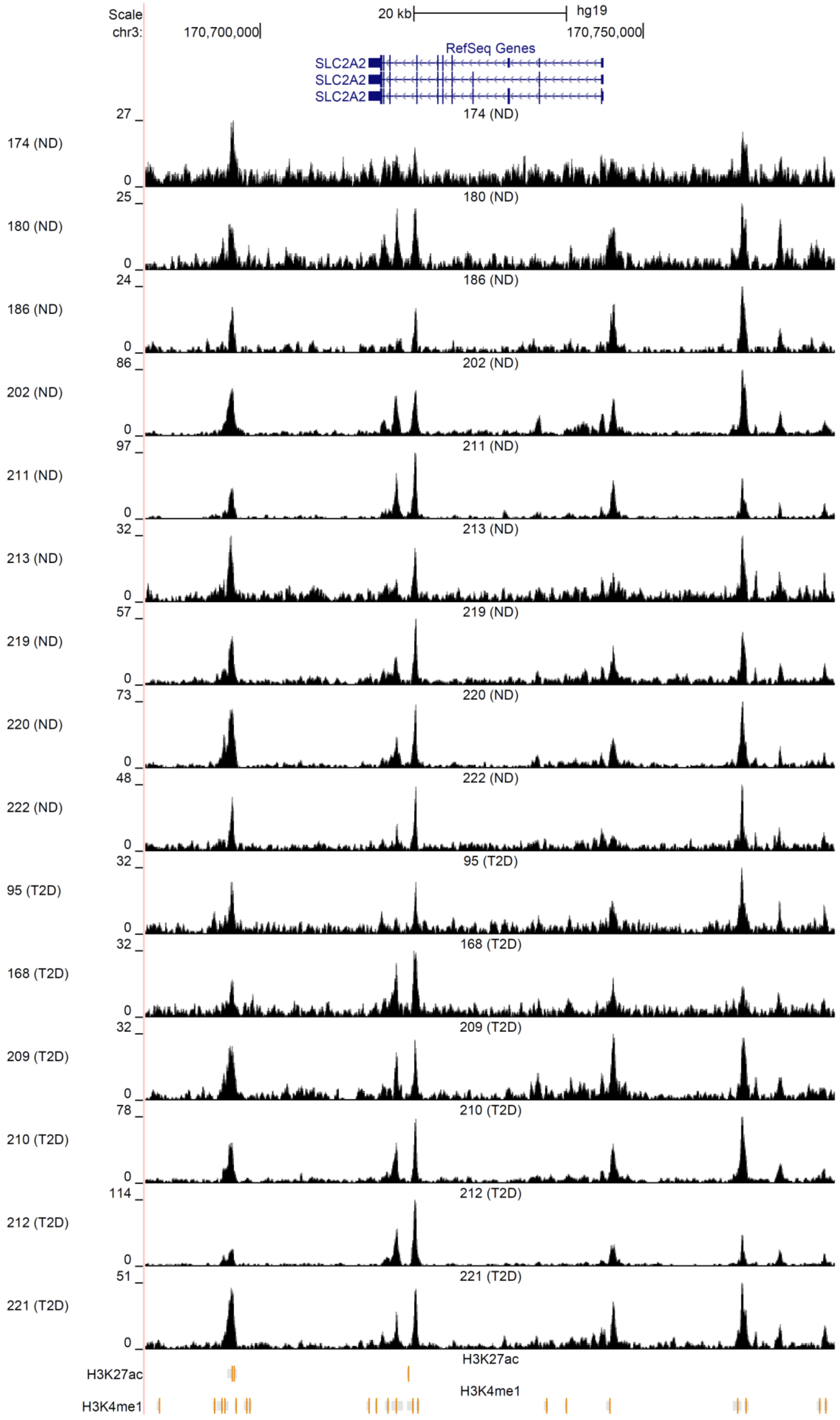

Supplemental Fig. 4 Representative sequencing tracks for the *SLC2A2* locus show ATAC-seq peaks that overlap with H3K4me1 and H3K27ac in human islets of all analysed donors.

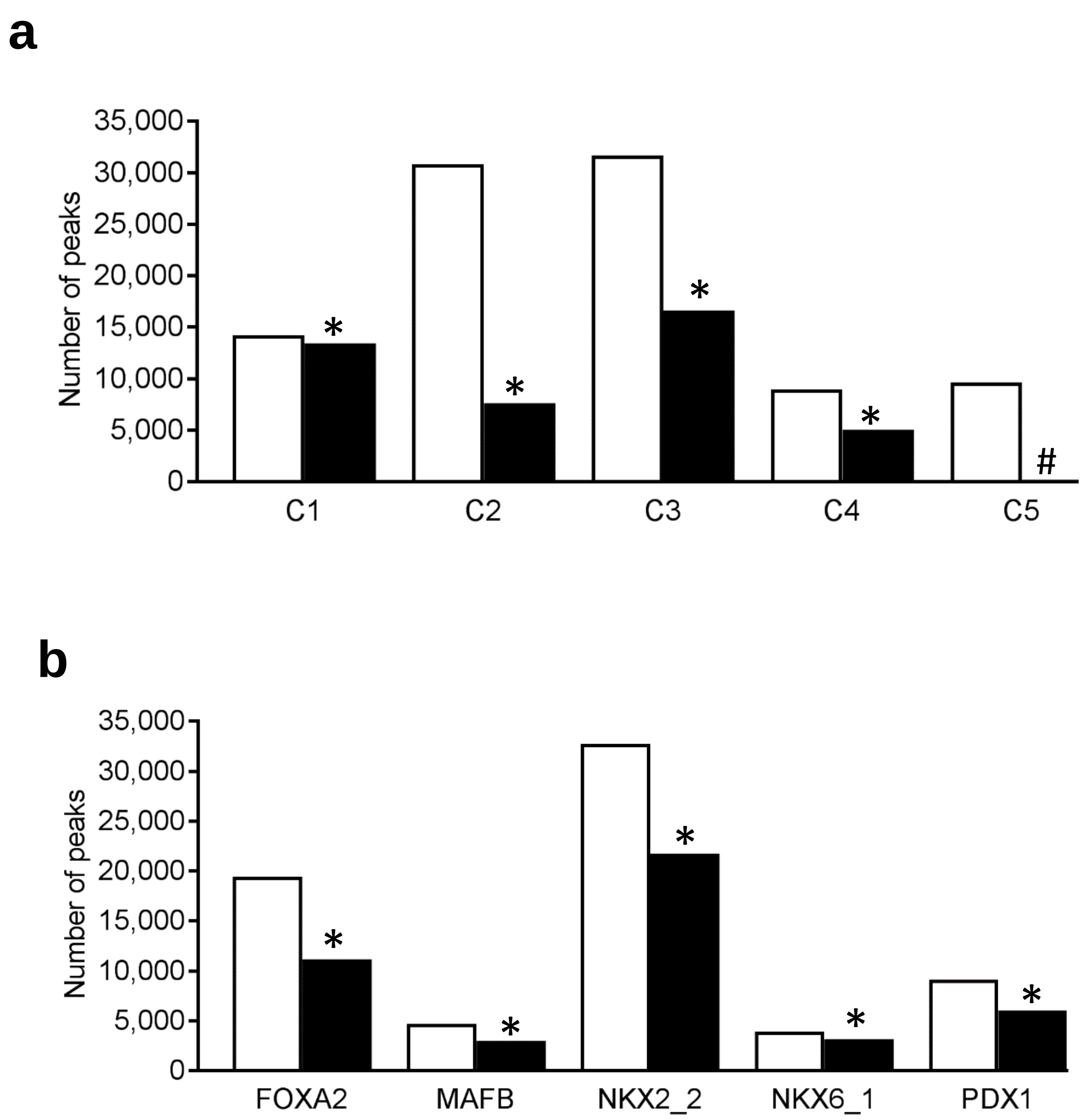

Supplemental Fig. 5 Bar graph of overlapping classified promoters (C1 sites), inactive enhancers (C2 sites), active enhancers (C3 sites), CTCF bound sites (C4 sites) and other sites (C5) and islet ATAC-seq peaks generated in (**a**) donors with T2D. White bars (Peaks) represent classified promoter regions generated by Pasquali et al. [11], black bars (Overlaps) represent ATAC-seq peaks that overlap with classified promoter regions. Based on chi-square tests there were significantly more ATAC-seq peaks than expected by chance overlapping with C1, C2, C3 and C4 (* $p<0.00001$, $q<0.01$) while significantly less than expected overlapping with C5 (# $p<0.00001$, $q<0.01$). Bar graph of overlapping transcription factor binding sites and islet ATAC-seq peaks generated in (**b**) donors with T2D. White bars (All sites) represent transcription factor binding sites classified by Pasquali et al. [11], while black bars (Overlaps) represent ATAC-seq peaks that overlap with transcription factor binding sites. The binding of all these transcription factors was enriched in islet ATAC-seq peaks with $p<0.001$ (* $q<0.018$). The classified promoters and transcription factor binding sites were generated by Pasquali et al. [11].

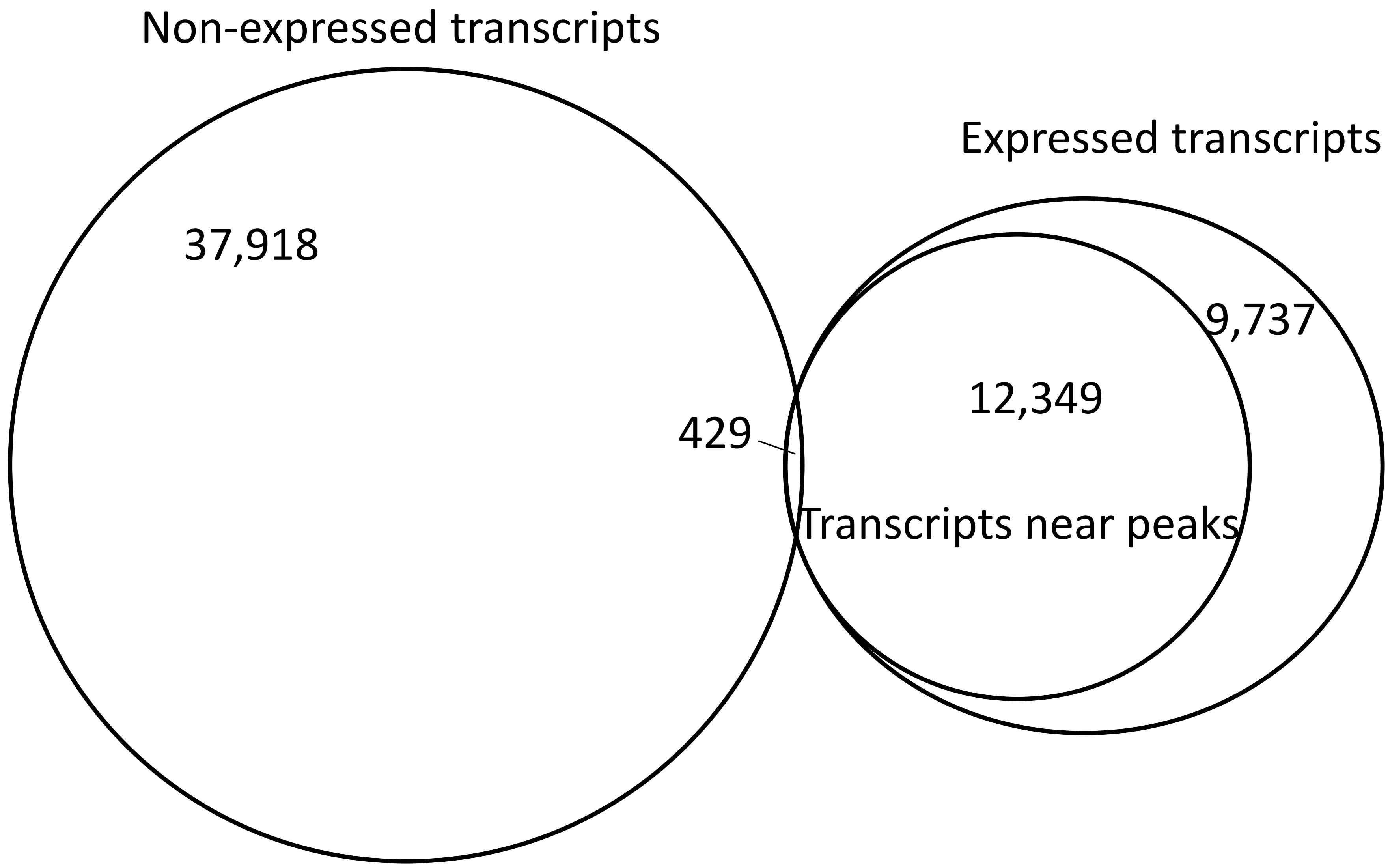

Supplemental Fig. 6 Venn diagram showing proportion of expressed and not expressed transcripts (transcript per million < 0.1) near ATAC seq peaks in human islets of non-diabetic donors. Genes were classified based on whether any of their annotated features (TSS, TTS, intron, exon, 3'UTR, 5'UTR) were within 1,500 bp of a peak summit. Only peaks which were found in ≥ 3 non-diabetic islet donors were taken into account. 37,918 transcripts were not expressed and not close to an ATAC-seq peak, 429 transcripts were not expressed and close to an ATAC-seq peak, 12,349 transcripts were expressed and close to an ATAC-seq peak and 9737 transcripts were expressed and not close to an ATAC-seq peak.

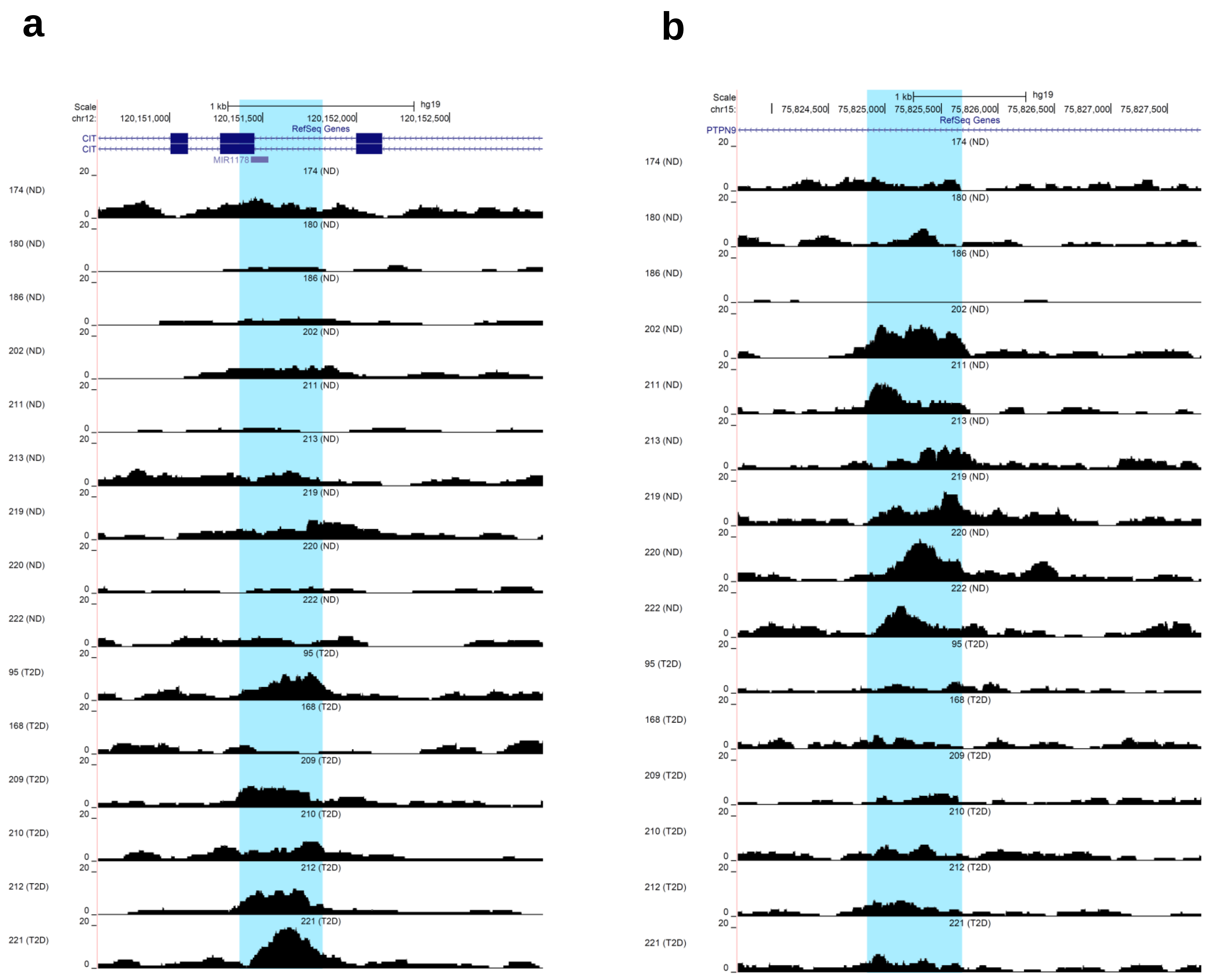

Supplemental Fig. 7 Sequencing tracks of ATAC-seq peaks that are more prevalent in donors with type 2 diabetes (annotated to *MIR1178*) (**a**) or non-diabetics (annotated to *PTPN9*) (**b**) of all studied islet donors. The ATAC-seq data have been normalized to take sequencing depth into account.

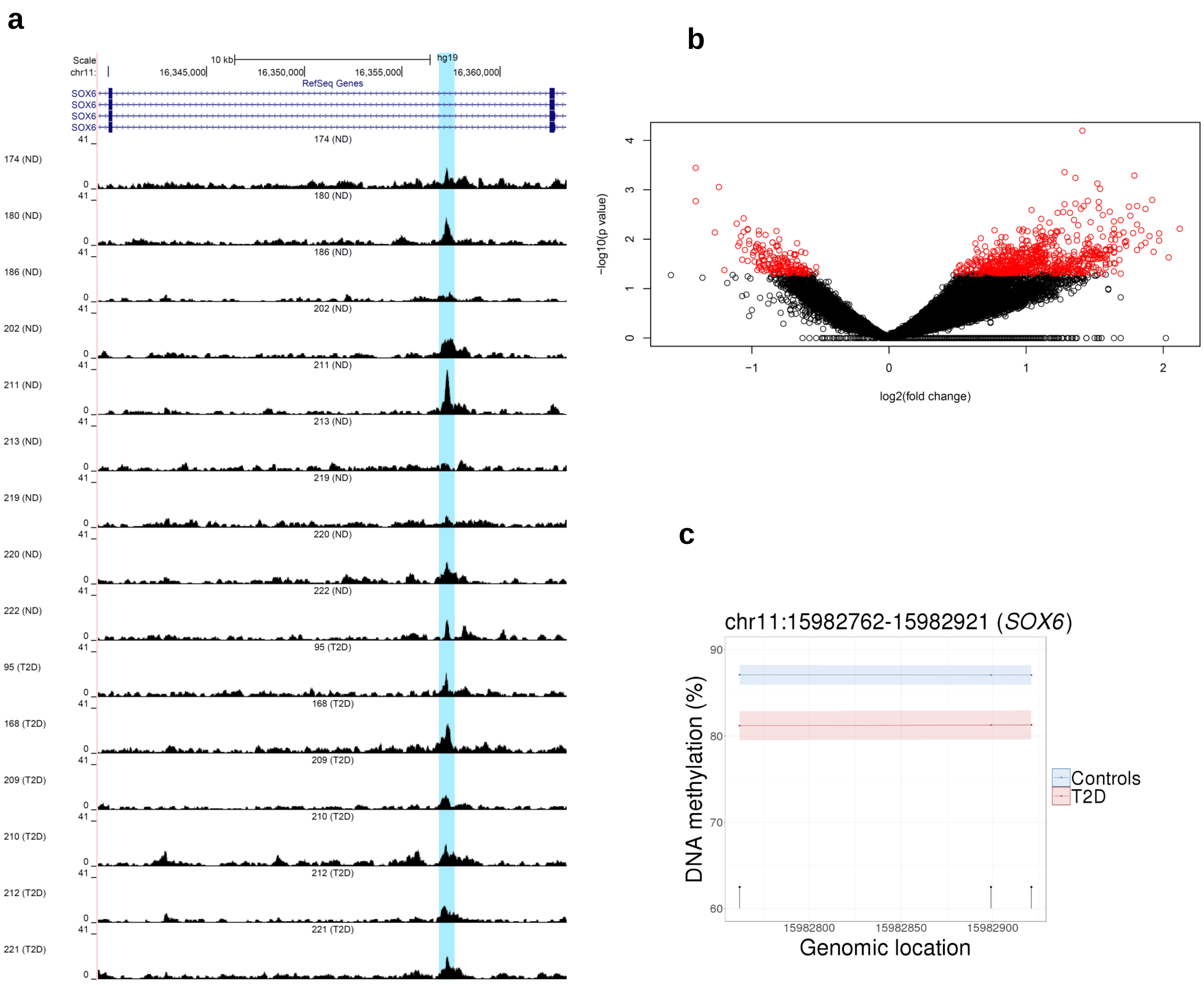

Supplemental Fig. 8 (a) Sequencing tracks of ATAC-seq peaks that are more prevalent in donors with type 2 diabetes (annotated to an intron of *SOX6*). (**b**) Volkano plot of human islet ATAC-seq data of donors with type 2 diabetes versus non-diabetic donors analysed with R Diffbind and edgeR packages (**c**) Type 2 diabetes associated differentially methylated region (DMR) annotated to *SOX6* and analysed by whole-genome bisulfite sequencing. Here, subjects with type 2 diabetes show lower degree of methylation compared to non-diabetic controls and these data have previously been published/presented in Supplementary Table 3 by P Volkov et al. (Diabetes 2017).

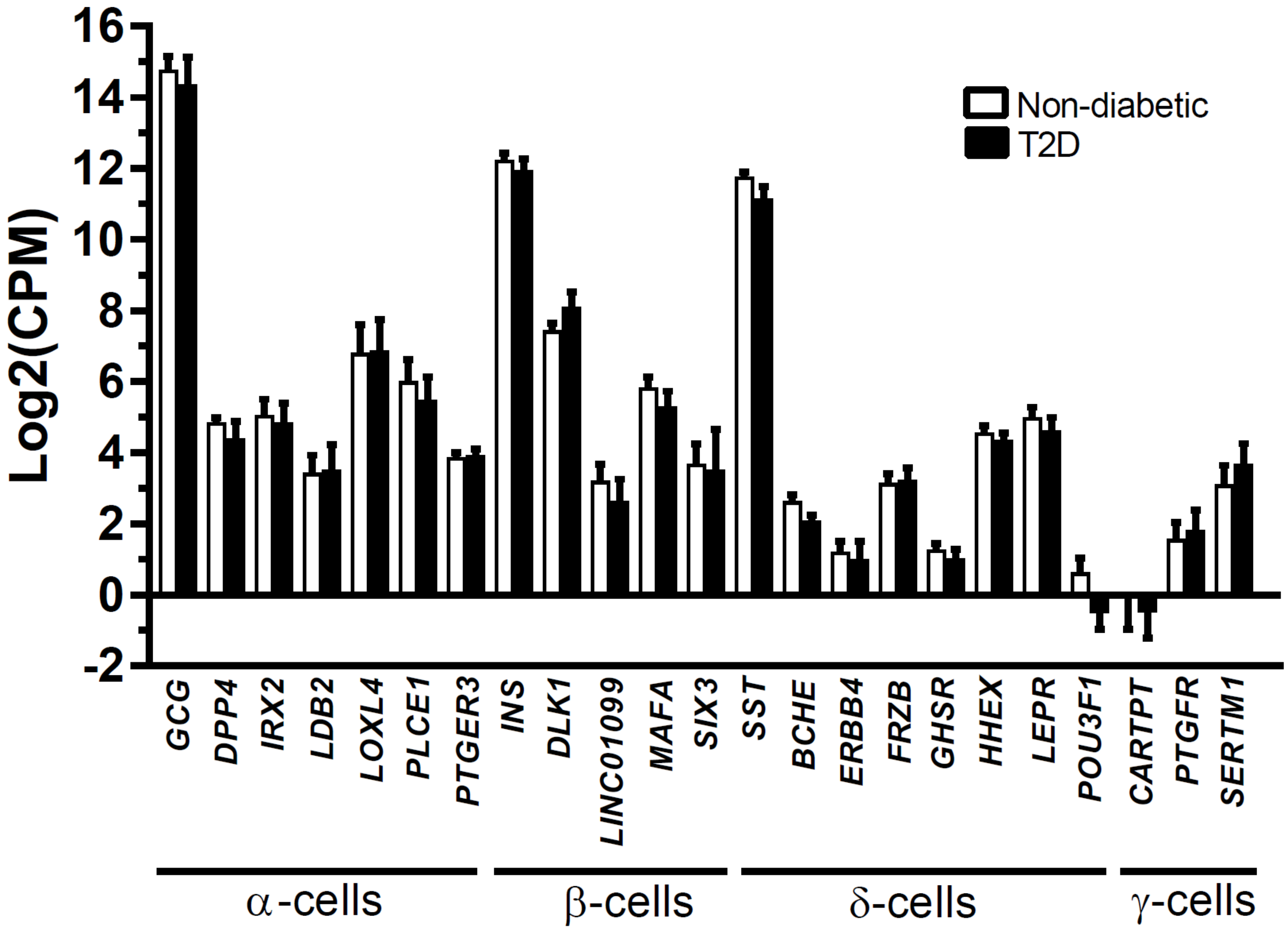

Supplemental Fig. 9 No significant differences in gene expression of some cell specific genes analyzed by RNA-seq in human islets from type 2 diabetic (T2D, n=5) versus non-diabetic (n=5) donors.
